# Peeling the onion: another layer in the regulation of insulin secretion

**DOI:** 10.1172/JCI169718

**Published:** 2023-04-17

**Authors:** Kristie I. Aamodt, Alvin C. Powers

**Affiliations:** 1 Department of Pediatrics, Division of Endocrinology, Boston Children’s Hospital, Boston, Massachusetts, USA.; 2 Islet Cell and Regenerative Biology Section, Joslin Diabetes Center, Boston, Massachusetts, USA.; 3 Department of Medicine, Division of Diabetes, Endocrinology and Metabolism, Vanderbilt University Medical Center, Nashville, Tennessee, USA.; 4 Department of Molecular Physiology and Biophysics, Vanderbilt University, Nashville, Tennessee, USA.; 5 VA Tennessee Valley Healthcare, Nashville, Tennessee, USA.

## Abstract

Insulin secretion by pancreatic β cells is a dynamic and highly regulated process due to the central importance of insulin in enabling efficient utilization and storage of glucose. Multiple regulatory layers enable β cells to adapt to acute changes in nutrient availability as well as chronic changes in metabolic demand. While epigenetic factors have been well established as regulators of chronic β cell adaptations to insulin resistance, their role in acute adaptations in response to nutrient stimulation has been relatively unexplored. In this issue of the *JCI*, Wortham et al. report that short-term dynamic changes in histone modifications regulated insulin secretion and acute β cell adaptations in response to fasting and feeding cycles. These findings highlight the importance of investigating whether other epigenetic mechanisms may contribute to acute physiologic adaptations in β cells.

## Importance of adaptive insulin secretion

In response to fluctuations in life-sustaining nutrient availability and consumption, humans and other animals have evolved complex, integrated metabolic pathways that allow an organism to respond dynamically by altering fuel metabolism (1). For example, during long periods of fasting or after greatly increased nutrient consumption, the blood glucose is maintained in a very narrow range. Insulin, secreted by the pancreatic β cell in response to a postprandial increase in plasma glucose, is the central anabolic hormone that promotes the utilization and storage of metabolic fuel in response to nutrient input. Insulin secretion is potentiated or modified by other fuels, including amino acids and fatty acids along with hormones and peptides produced in pancreatic islets and the gastrointestinal tract. The effects of insulin are opposed by glucagon that drives mobilization and oxidation of metabolic stores during fasting or hypoglycemia.

**Figure 1:**
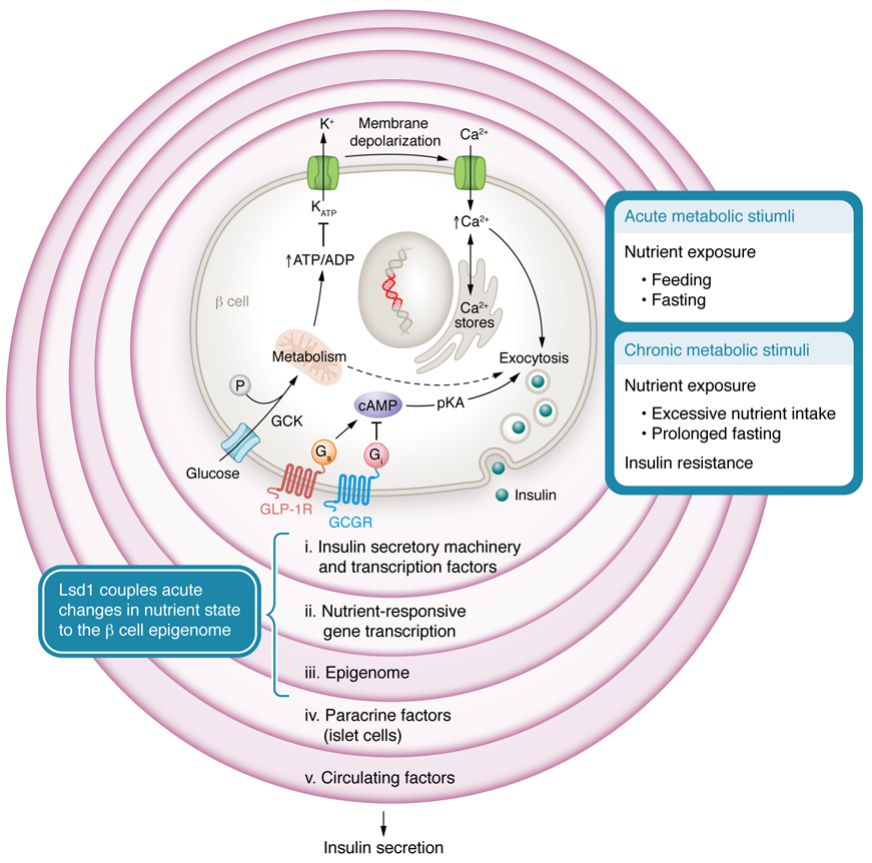
Complex interactions across multiple regulatory layers generate dynamic changes in insulin secretion in response to acute and chronic metabolic stimuli. (i) The insulin secretory mechanism intrinsic to β cells regulates the triggering phase of GSIS, where an increase in plasma glucose rapidly activates insulin secretion. Glucose metabolism generates ATP through mitochondrial oxidative metabolism and additional noncanonical pathways (dotted line) (8). This increase in intracellular ATP closes K_ATP_ channels, which depolarizes the membrane, consequently activating voltage-gated Ca^2+^ channels. Subsequent increases in intracellular Ca^2+^ trigger exocytosis of insulin secretory granules. Several mechanisms, predominantly K_ATP_ channel independent, potentiate insulin secretion during the amplifying phase via intermediary metabolites and metabolic coupling factors that further increase trafficking and secretion of insulin granules. (ii) Initiation of GSIS activates transcriptional changes in nutrient-responsive genes, allowing for β cell adaptation to acute cycles of feeding and fasting. The β cell adapts to prolonged nutrient stimulation by increasing proinsulin synthesis and compensatory hypertrophy of the ER and Golgi complex. (iii) Wortham et al. (7) identified a role for Lsd1-mediated changes of histone acetylation in regulating the expression of these nutrient-responsive genes. Other epigenetic regulators of insulin secretion, including DNA methylation, RNA modifications, and ncRNAs, play a role in chronic β cell adaptations to insulin resistance. (iv) Paracrine signals from α cells, such as glucagon, GLP-1, and acetylcholine, potentiate insulin secretion, while δ cells counterbalance GSIS via somatostatin. Glucagon and GLP-1 work through G protein–coupled receptors (GCGR and GLP-1R) to activate cAMP/pKA signaling. (v) Insulin secretion is also regulated acutely in response to circulating factors that are increased with feeding. Nutrients, including amino acids and FFAs, along with circulating incretin hormones from the gastrointestinal tract, including GLP-1 and GIP, promote insulin secretion and acute β cell adaptations. Specific circulating miRNAs have also been shown to regulate insulin secretion and chronic β cell adaptations.

Although the basic mechanisms driving glucose-stimulated insulin secretion (GSIS) are relatively well established, multiple layers of regulatory inputs potentiate, dampen, or otherwise modify this process (2). This regulatory complexity is not unexpected given that a careful balance is needed to maximize fuel storage and utilization in response to changing states of nutrient availability and metabolic demand. Mechanisms within β cells and external to β cells drive adaptations in response to nutrient input to ensure that insulin production and secretion appropriately match metabolic demand (Figure 1) (3). These β cell adaptations are particularly important when insulin demands or insulin sensitivity change, such as with pubertal growth, pregnancy, obesity, or insulin resistance (4, 5). Type 2 diabetes (T2D) develops when β cells are unable to adapt to this increased demand by sufficiently increasing insulin secretion.

Epigenetic regulation of β cell adaptations that take place as a chronic response, such as with insulin resistance or increased fat consumption, has been widely explored due to implications for diabetes (6). These adaptations occur over a period of weeks or months and often involve changes in β cell function, β cell number, and/or β cell mass. In this issue of the *JCI,* Wortham et al. identify a role for epigenetic regulatory mechanisms in acute physiologic β cell adaptation to nutrient stimulation, highlighting the need for better characterizing the layers involved with dynamically regulating the acute and chronic β cell response to physiologic inputs (Figure 1) (7).

## Acute regulation of insulin secretion

Glucose is the primary stimulus for insulin secretion. During the initial triggering phase, elevated plasma glucose levels result in rapid release of insulin granule contents (Figure 1). The initial steps of this process involve facilitated glucose transporters and glucokinase (GCK), with GCK as the rate-limiting step in insulin secretion. Subsequently, ATP, generated from glucose metabolism, closes ATP-sensitive potassium (K_ATP_) channels, which depolarizes the membrane to activate voltage-gated Ca^2+^ channels. The consequent rapid rise in intracellular Ca^2+^ levels activates exocytosis of readily releasable insulin granules. Canonical models of GSIS center on oxidative glucose metabolism as the dominant metabolic driver of insulin secretion. However, these models do not adequately capture the complex interplay among the numerous metabolic cycles, coupling factors, and spatially distinct metabolic compartments, including mitochondrion, cytosol, and the area beneath the plasma membrane, required to achieve, sustain, and terminate GSIS (8). Insulin secretion is further potentiated during the amplifying phase by a variety of mechanisms that promote insulin granule exocytosis but are predominantly K_ATP_ channel independent (2). Several of these potentiating mechanisms include the accumulation of intermediary metabolites, such as citrate and malate. Other potentiating processes are activated by exposure to other nutrients, including amino acids and free fatty acids (FFAs). Several β cell–specific genes and transcription factors that encode and/or regulate components of this insulin secretory pathway have been implicated in monogenic and neonatal forms of diabetes (9).

The pancreatic islet is a mini-organ, and paracrine signaling between β cells and other islet cells plays an important role in modulating GSIS (10). Signals from α cells potentiate insulin secretion through exocytosis of insulin granules. Specifically, glucagon and glucagon-like peptide 1 (GLP-1) released from α cells activate cAMP, which mediates exocytosis of insulin granules by direct actions on secretory granule trafficking and exocytosis and by increasing intracellular Ca^2+^. Acetylcholine is also released from α cells and, upon binding to muscarinic receptors on β cells, leads to increased intracellular Ca^2+^ and subsequent exocytosis of insulin granules. GLP-1 along with glucose-dependent insulinotropic polypeptide (GIP) and other hormones, sometimes termed incretins, are released from the gastrointestinal tract and also promote cAMP-mediated insulin release (11). Islet δ cells, after integrating multiple inputs and counterbalances, release somatostatin to negatively regulate insulin secretion.

In response to ongoing nutrient exposure during this period of sustained insulin release, β cell secretory capacity begins to adapt to ensure ongoing insulin availability (12). Substantial changes in the β cell transcriptome accompany this response to nutrient exposure. Wortham et al. used a mouse model of time-restricted feeding and demonstrated that histone acetylation changes in islets regulate these β cell adaptations on a much shorter timescale than previously suspected. The authors observed that β cell functional adaptations in these mice were apparent within four hours after feeding. Changes in histone H3 lysine 27 acetylation (H3K27ac) were required for the appropriate expression of nutrient-induced genes in response to both fasting and feeding. Notably, the chromatin-modifying lysine-specific demethylase-1 (Lsd1) regulated these changes. Lsd1 was recruited to sites in the fasted state, and these sites gained H3K27ac with feeding. These results suggest that Lsd1 acts as a dynamic regulator of chromatin accessibility that is responsive to short-term changes in nutrient state during feeding and fasting cycles (7).

β Cell–specific knockout of *Lsd1* in mice led to dysregulated expression of these nutrient-responsive genes and most notably caused insulin hypersecretion resulting in hypoglycemia. These mice lost their ability to suppress insulin secretion during fasting and had profound hypersecretion in response to feeding along with an exaggerated response to the GLP-1 analog exendin-4. This dysregulation was due to inappropriate activation of both triggering and amplifying pathways. Similar effects were seen in human islets in which inhibition or knockdown of LSD1 resulted in transcriptional changes in nutrient-responsive gene expression and increased basal insulin secretion (7). These findings suggest that the regulation of chromatin accessibility via histone modifications plays an important role in the process of fine-tuning insulin secretory response in order to maintain glucose homeostasis.

## Chronic β cell adaptations in sustained insulin secretion

Chronically stimulated β cells can expand secretory capacity to maintain sustained insulin secretion in response to ongoing excessive nutrient exposure. Compensatory strategies to achieve this expanded capacity include increasing proinsulin synthesis via increased transcription, mRNA stabilization, translation, and hypertrophic expansion of the endoplasmic reticulum and Golgi complex. Multiple epigenetic mechanisms mediate these chronic β cell adaptations to insulin resistance, which are overwhelmed in T2D (6). Regulation of chromatin accessibility through DNA methylation and histone modifications is critical to β cell function and adaptation to insulin resistance, and aberrant methylation patterns of genes regulating these processes are associated with T2D. The importance of posttranscriptional N6-methyladenosine (m^6^A) placement on key β cell transcripts is also critical for β cell compensation and GSIS; hypomethylation of these transcripts is seen in islets from T2D patients (13). A wide range of noncoding RNAs (ncRNAs) also have regulatory roles in β cell function and adaptation. Specific miRNAs are involved with regulating insulin synthesis, secretion, and T2D pathogenesis (14), and small nucleolar RNAs (snoRNAs) have noncanonical functions in GSIS regulation in mice (15, 16).

Using an insulin-resistant mouse (db/db) as a model of chronic β cell adaptation, Wortham et al. found overlap with genes upregulated in acute β cell response to feeding along with hyperacetylation of H3K27 at many of the feeding-induced sites identified previously. The authors also performed ChIP-Seq for LSD1 in human islets and found that the LSD1-bound active chromatin was enriched for T2D-associated risk variants, demonstrating a connection for Lsd1 in chronic as well as acute β cell adaptation (7).

## Implications and future directions

The observation by Wortham et al. (7) that epigenetic regulation of insulin secretion in both mouse and human islets occurs on an acute timescale, rather than solely as a chronic response to insulin resistance, provides important insight into the dynamic role that epigenetic regulators can play in acute physiologic β cell adaptations. This discovery brings important questions to the forefront about how other epigenetic regulatory mechanisms known to be altered in response to chronic insulin resistance may also be modulating β cell adaptations more acutely in response to fasting and/or feeding. Integration of these findings with noncanonical models of GSIS are also needed. And while Wortham et al. (7) identified a link between the LSD1-mediated modifications in response to nutrient signals and those noted from insulin-resistant mice, these changes were not identical, suggesting that additional regulatory mechanisms are involved (7). Moving forward, it will be essential to determine to what degree epigenetic mediators of acute insulin secretion overlap with those regulating chronic β cell adaptations. Given the importance of paracrine signaling in the regulation of insulin secretion, it will also be important for future studies to explore the role epigenetics may have on altering α and δ cell function and the subsequent effects on insulin secretion. This work provides important insight into how dynamic epigenetic regulators of insulin secretion can mediate the response to acute changes in nutrient signaling. As future studies further unravel the regulatory layers connecting these acute epigenetic changes to chronic epigenetic alterations in response to insulin resistance, we will gain critical insight into how these mechanisms relate to increased or decreased nutrient intake and insulin dysregulation.

## Version 1 04/17/2023

Electronic publication
